# Numerical Simulation of Fatigue Crack Growth and Fracture in Welded Joints Using XFEM—A Review of Case Studies

**DOI:** 10.3390/ma17225531

**Published:** 2024-11-13

**Authors:** Aleksandar Sedmak, Aleksandar Grbović, Nenad Gubeljak, Simon Sedmak, Nikola Budimir

**Affiliations:** 1 Faculty of Mechanical Engineering, University of Belgrade, 11000 Belgrade, Serbia; asedmak@mas.bg.ac.rs (A.S.); agrbovic@mas.bg.ac.rs (A.G.); 2 Faculty of Mechanical Engineering, University of Maribor, 2000 Maribor, Slovenia; nenad.gubeljak@um.si; 3 Innovation Center of Faculty of Mechanical Engineering, 11100 Belgrade, Serbia

**Keywords:** fatigue crack growth, extended finite element method, welded joints, fatigue life, high-strength low-alloyed steel

## Abstract

Numerical simulation of fatigue crack growth in welded joints is not well represented in the literature, especially from the point of view of material heterogeneity in a welded joint. Thus, several case studies are presented here, including some focusing on fracture, presented by two case studies of mismatched high-strength low-alloyed (HSLA) steel welded joints, with cracks in the heat affected zone (HAZ) or in weld metal (WM). For fatigue crack growth, the extended finite element method FEM (XFEM) was used, built in ABAQUS and ANSYS R19.2, as presented by four case studies, two of them without modelling different properties of the welded joint (WJ). In the first one, fatigue crack growth (FCG) in integral (welded) wing spar was simulated by XFEM to show that its path is partly along welded joints and provides a significantly longer fatigue life than riveted spars of the same geometry. In the second one, an integral skin-stringer panel, produced by means of laser beam welding (LBW), was analysed by XFEM in its usual form with stringers and additional welded clips. It was shown that the effect of the welded joint is not significant. In the remaining two papers, different zones in welded joints (base metal—BM, WM, and HAZ) were represented by different coefficients of the Paris law to simulate different resistances to FCG in the two cases; one welded joint was made of high-strength low-alloyed steel (P460NL1) and the other one of armour steel (Protac 500). Since neither ABAQUS nor ANSYS provide an option for defining different fatigue properties in different zones of the WJ, an innovative procedure was introduced and applied to simulate fatigue crack growth through different zones of the WJ and evaluate fatigue life more precisely than if the WJ is treated as a homogeneous material.

## 1. Introduction

Welded joints are well known for their efficiency in joining metals, but also for their susceptibility to cracking and heterogeneity of microstructure, especially in the case of HSLA steel, leading to different properties in different zones (Base Metal—BM, Weld Metal—WM, and Heat Affected Zone—HAZ), including crack and fracture resistance. It is well known that the WM and HAZ commonly have lower fracture resistance compared to BM, as shown in [1,2,3] in the case of HSLA steels and SA-387 Gr. 91 steel, respectively, and an even more pronounced resistant with respect to fatigue crack growth, as shown in [4] for SA-387 Gr. 91 steel.

Regarding quality and design requirements, which are of great importance when it comes to welded structures, different case studies presented here covered different quality classes for welds. These included welded joints in aircraft parts, which require the highest quality class according to EN-ISO-5817 [5]. Relevant standards were also used for other case studies, including, for example, EN 13445 [6], which was considered for cases involving pressure vessels, and EN 13480 [7], which was used for pipelines. The design of welded structures is also important since inadequate geometry could make it difficult to weld and later control the joints. Numerical analyses are useful in such cases, since they provide some insight into the behaviour of these inaccessible welds. In addition, design requirements often involve welding of dissimilar steels, which can also be effectively simulated using FEM, as shown in [8,9].

There is a large number of papers dealing with the fracture of welded joints, with a focus on the mismatching effect, both in the case of a stronger weld metal (overmatching) or a stronger base metal (undermatching) [10,11,12,13,14,15,16]. A very useful and practical approach to analyse the mismatching effect was introduced in the 1990s, as presented in [10], leading to the IIW document, ref. [6], and the SINTAP procedure described in [12,13,14,15,16,17]. A more detailed analysis of the effects of welded joint strength mismatching on crack driving force is given in [16] from the point of view of stress and strain-based design. Mismatching was also analysed experimentally and numerically in two papers dealing with overmatching [17], and undermatching [18], which will be presented later in this paper as two case studies. One should notice the following two opposite arguments for practical applications: one approach is designed to use overmatching to shift eventual plastic deformation and damage, i.e., cracking, to the BM, which is more resistant to crack growth; whereas the other one is designed to use softer filler metal to obtain weld metal sufficiently resistant to cracking. This issue was also covered in papers [19,20]. Fracture mechanics analysis of the surface crack tips in heat affected zones showed that the overmatching effect caused a redirection of the crack propagation towards the low-strength region. It was also shown that even in the case of a soft root layer, it was possible to achieve satisfying crack resistance and avoid preheating at the same time [19]. Yet, another aspect of this problem was presented in [20], where the constraint effect on fracture behaviour in strength-mismatched weld joints was analysed. It was found that the width of the welded joint as a constraint parameter has an effect on the limit load and maximum load, and it achieved fracture toughness values equal to those of a welded joint. 

Finally, in this analysis of static behaviour of mismatched welded joints, it is important to briefly tackle the problem of determination of tensile properties, i.e., stress–strain curves of different zones of welded joints, including editorial paper [21]. In a couple of recent papers [22,23], this problem was solved by combining efforts of experimental research using DIC to measure strains along the welded joint and its numerical simulation by FEM, with an iterative procedure to match the experimental results. 

Another important issue of WJ behaviour, both under static and dynamic loading, is the effect of residual stresses. Numerical simulation of welding treated as a thermomechanical process, verified by experimental research, was presented in [24] as a methodology based on using SYSWELD. Later on, its application with a focus on residual notch stress intensity factor evaluation was presented in papers [25,26]. 

Even more critical for welded joint behaviour is fatigue amplitude loading, regular or irregular, which is the most common cause of premature failures. Fatigue is a complex phenomenon, based on crack growth in a specific way, with several parameters which are not easy to follow (stress amplitude ratio, fatigue crack propagation threshold ΔK_th_, and material heterogeneity, just to mention a few of them [27]). It is still more a descriptive than a deterministic process, but ever since Paris published his famous empirical law [28], there is at least sound basis to analyse fatigue crack growth (FCG) both experimentally and numerically. 

Experimental evaluation is focused on the determination of the Paris law coefficients, *C* and *m*, defined by exponential relation between crack growth rate and the stress intensity range:

(1) $$\frac{da}{dN}=C\cdot {\left(\Delta K\right)}^{m}$$

Procedure for determination of *C* and *m* is well defined by standard [29].

Numerical simulation of fatigue was almost impossible before the 1990s because the classical FEM approach required the successive generation of a mesh in each step of crack growth, as described in the review paper [30]. To overcome this problem, the XFEM was introduced [31,32,33], enabling crack propagation modelling without the modification of the FE mesh. This was achieved by using additional approximation, i.e., enrichment functions, to model a crack by additional degrees of freedom to the nodes which are crossed by the crack. In its essence, XFEM allows for cracks to arbitrarily cut through finite elements, providing not only a mesh-free method, but also an option to predict the crack growth path. Generally speaking, the case studies presented here proved that XFEM is a powerful tool in solving engineering problems related to the FCG. 

One should keep in mind that XFEM was introduced to enable the relatively simple modelling of crack growth, since it does not require re-meshing. For about two decades, re-meshing was so complicated that classic FEM was practically not used for crack growth simulation. This has changed in recent years, after SMART was introduced by ANSYS, allowing the automatic re-meshing process and making FEM an equally powerful tool in solving fatigue problems. In any case, fatigue crack growth in welded joints is not well presented in the literature, especially from the point of view of their heterogeneity.

Therefore, in this paper, XFEM is applied to solve practical problems, as presented in the form of four additional case studies related to welded joints, two of them from early developments without modelling of different zones in WJ [34,35], and the other two from more recent research on welded joints, including innovative procedures to model all zones in the WJ, since neither ABAQUS nor ANSYS provide that option yet [36,37]. Regarding static fracture, two case studies are presented to illustrate the effect of material heterogeneity in overmatched and undermatched welded joints, including the fine grain and coarse grain HAZ.

One should notice that the welded joint geometries considered here involve butt-welded joints, which are the most commonly used type of joints in pressure vessels and pipelines, and fillet welds in the case of aircraft components. 

## 2. Case Study 1—Overmatching Effects on Crack Growth Through HAZ

**Figure 1:**
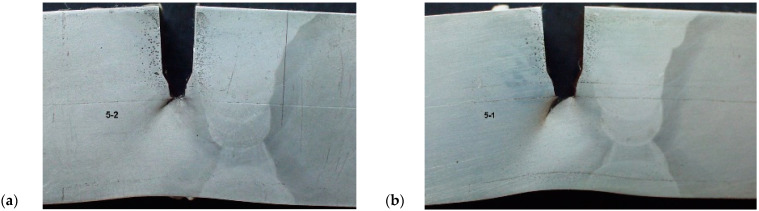

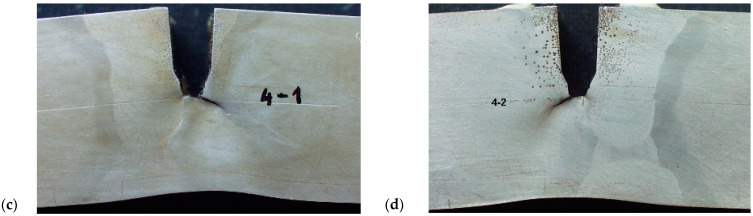
Crack tip location and growth toward the BM: (**a**,**b**) FG HAZ, (**c**,**d**) CG HAZ [17].

The overmatching effects on the static crack growth through the heat affected zone of the HSLA steel welded joint were analysed both experimentally and numerically, as presented in [17]. The steel used in this research was HSLA with a yield strength close to 500 MPa, welded by the manual metal arc process, as shown in [17]. Experiments were performed on tensile panels, considering the fact that different tensile properties in the BM, WM, and HAZ (fine grain—FG, and coarse grain—CG), as well as the crack tip location, dictate the elastic–plastic zone of crack growth, as described in more details in [17]. Two different crack tip locations were analysed, one in the fine grained HAZ (FG HAZ) and the other one in the coarse grained HAZ (CG HAZ). In both cases, after initial growth through the FG HAZ or CG HAZ, respectively, cracks were directed toward a more ductile base metal, thus increasing the fracture resistance of the welded joint, Figure 1. 

**Figure 2:**
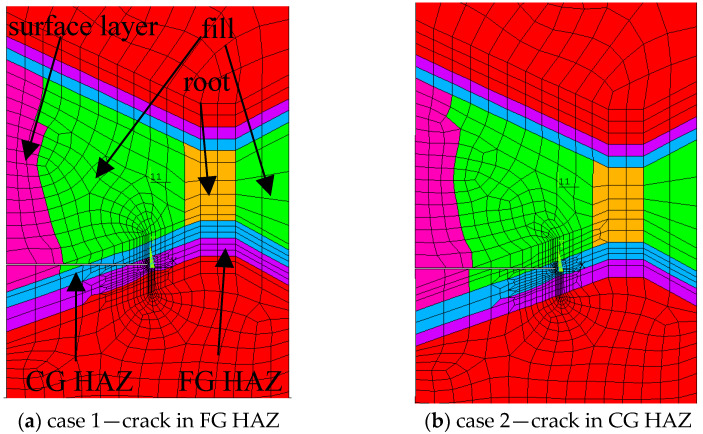
The simplified FEM model of welded joints—fine-grained HAZ (**a**) and coarse-grained HAZ (**b**) [17].

Tensile specimens were also analysed numerically by the finite element method, requiring a large number of 3D 20-node elements (4560) in ANSYS 5.7 code, as shown in more details in [17]. Special care was taken to model the HAZ and crack region, as shown in Figure 2 for both cases of crack tip location. Comparison of numerical and experimental results for J vs. CMOD and F vs. CMOD curves indicated good agreement, as shown in [17]. 

**Figure 3:**
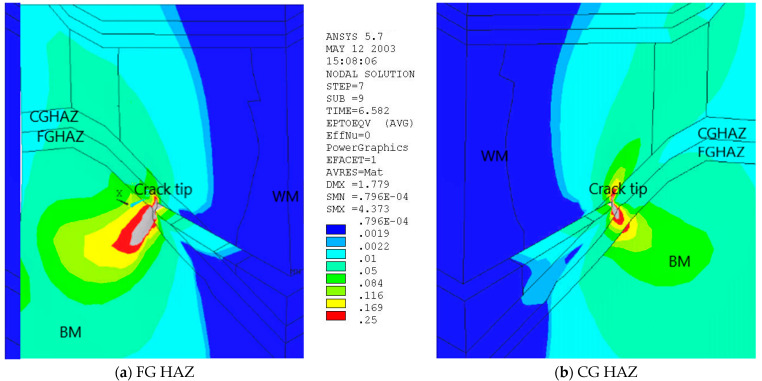
Equivalent strain distributions for the remote stress 292 MPa, for fine-grained HAZ (**a**) and coarse-grained HAZ (**b**) [17].

Strain distribution, obtained by 3D FEM at the level of remote loading close to 300 MPa, is shown in Figure 3 for both crack tip locations. More details about strain distribution can be found in [17]. The overmatching effect is obvious, acting as a barrier for plastic strain in both cases, shifting it to the more ductile base material and thus protecting the welded joint from failure. More concretely, for the case where the crack tip is located in the FG HAZ, the CG HAZ acts as a constraint, providing unfavourable triaxial stress conditions, whereas in the case where the crack tip is in the CG HAZ, plastic strain is directed to the more ductile WM and in the FG HAZ. Further on, in both cases, after the initial crack growth, even relatively small overmatching changed its direction toward a weaker and more ductile BM. 

Based on the briefly presented results, one can conclude that the overmatching in the welded joint has acted as a protective in respect to crack growth in both cases of crack tip location (FG HAZ or CG HAZ), increasing the overall fracture resistance. 

## 3. Case Study 2—Undermatching Effects on Fracture Behaviour of a Welded Joint

As shown in Case Study 1, strength overmatching in the welded joint with a crack in the HAZ is beneficial from the point of view of fracture behaviour due to crack growth redirection toward more resistant BMs, making this option of mismatching a good design solution for certain types of HSLA steels. Nevertheless, one should keep in mind that HSLA steels’ sensitivity to cracking increases with an increasing level of strength, so the undermatching welded joint is potentially a better design option in the case of YS above 700 MPa [18]. On the other side, this issue is not as simple as just avoiding cracking, since eventual plastic strain (due to stress concentration and low YS) could jeopardise the integrity of a WM, before a BM starts to yield [18]. Therefore, in this case study, the undermatching effects on the fracture behaviour of HSLA steel (SUMITEN SM 80P) welded joints were analysed experimentally and numerically, using tensile panels with the so-called small and large crack in the WM, as shown in more details in [18]. The Finite Element Method (FEM) with different tensile properties in BMs and WMs was used for the numerical simulation of tensile testing. Keeping in mind the importance of the material used in this analysis, the chemical composition and tensile properties of both BM and WM are presented here, Table 1 and Table 2, respectively, as well as the basic data about welding. From the data in Table 2, one can see that the undermatching ratio for Shielded Manual Arc Welding (SMAW) was 0.91, and 0.95 for Submerged Arc Welding (SAW). The basic coated low hydrogen electrode LB 118 for MAW and core wire US 8013 with M38F flux for SAW, “Cobe Steel”, Japan, were used. All other data and details are presented in [18].

**Figure 4:**
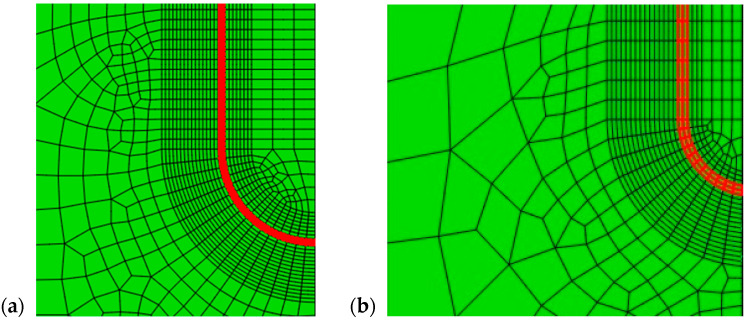
Geometry of numerically representation: (**a**) LSC, (**b**) SSC. Red lines mark crack front [18].

Three-dimensional FE models were developed to simulate the behaviour of the tensile panels with the SSC and LSC in the WM or in the BM. The effect of the crack tip fields due to mismatching were carefully studied using Abaqus, as described in more details in [18]. Here, a section of the FE mesh showing the LSC and SSC, is presented in Figure 4. The load was applied in the form of internal pressure which corresponded to the test pressure used during the testing of the real tank and was equal to 12.05 MPa.

**Figure 5:**
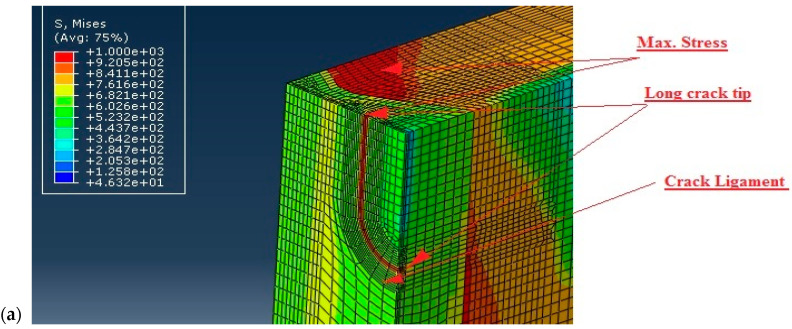

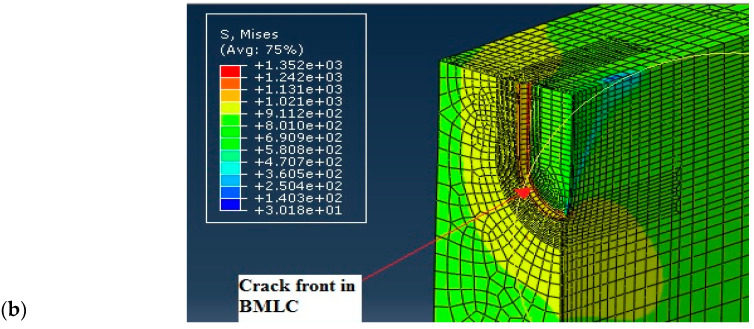
Comparison of the effects of LSC tip stress fields in (**a**) WM, (**b**) BM. Stresses in MPa [18].

**Figure 6:**
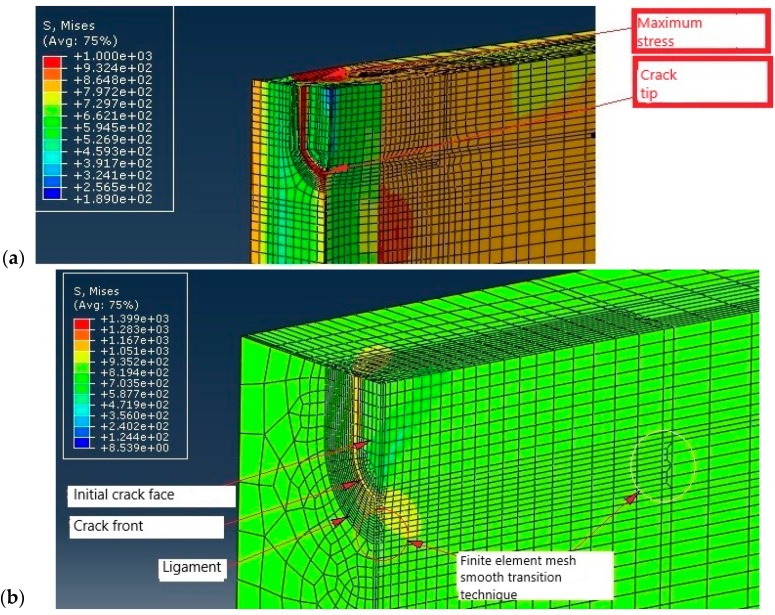
Comparison of the effects of SSC tip stress fields in (**a**) WM, (**b**) BM. Stresses in MPa [18].

Results are shown in Figure 5 indicating the von Mises stress distribution in the WM and BM for the LSC, and in Figure 6 for the SSC in the same way. 

As shown in Figure 5, there is a significant difference in stress distribution around the LSC in the WM and BM. One can see that the maximum stress in the BM is located at the crack tip, whereas the maximum stress in the WM is not only at the crack tip, but also in the BM. Such a redistribution of stresses and strains is beneficial for welded joint resistance to cracking, since it increases the crack resistance of the WM. The same comparison holds for the SSC, Figure 6, with the same consequences regarding the undermatching effect. Nevertheless, one should keep in mind that in both cases, in the SSC and LSC, the undermatching effect is beneficial only if the WM is capable of sustaining at least a small amount of plastic strain, which is the case here, as shown in Table 2, since the WM strain is over 20%.

One should notice that in both cases, under- and overmatching effects are favourable for fracture behaviour since the BM acts as a barrier to crack growth. Actually, heterogeneity in this case is beneficial, since a welded joint behaves better when the WM and/or the HAZ would behave as homogeneous structures. Nevertheless, these conclusions are limited to those regarding cracks in the HAZ of an overmatched WJ and cracks in a WM with an undermatched WJ.

Now, the attention shifts to amplitude loading and fatigue problems, as described by the following four case studies.

## 4. Case Study 3—The Effects of Welded Clips on Fatigue Crack Growth in AA6156 T6 Panels

**Figure 7:**
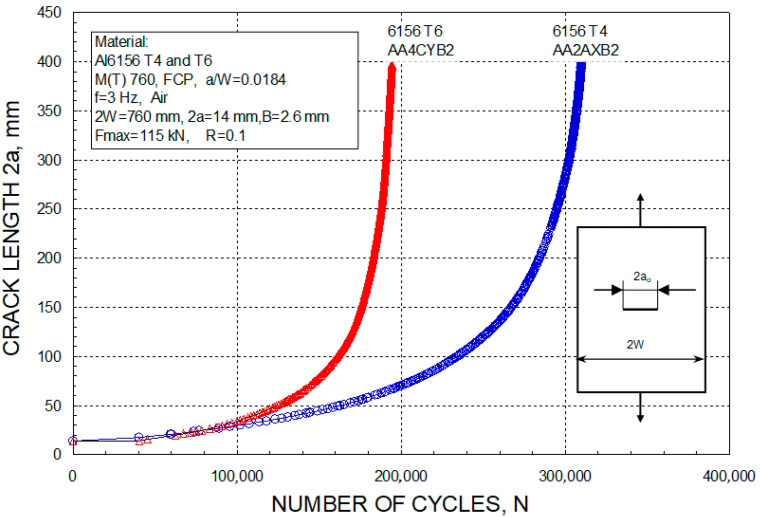
Load, stress ratio, and experimentally obtained numbers of cycles for the BM plate.

**Figure 8:**
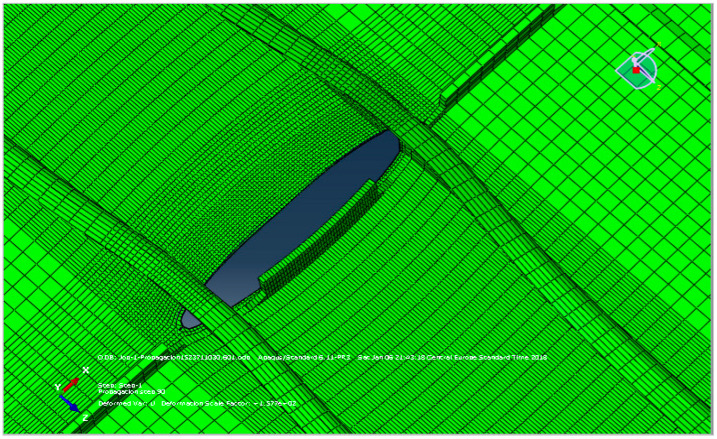
Central crack after 91 steps of growth [34].

As a part of large project performed almost two decades ago [34,38,39], fatigue crack growth in different panels, made of AA6156 T6, was investigated experimentally and numerically by XFEM. Three different types of panels were used, a simple one, the so-called base metal (BM), and then two panels with four welded stringers, one of them with three additional welded clips. The results of the experimental testing showed the significant improvement in the residual life of panels due to welded stringers, since the number of cycles were more than double compared with the base metal (422 Kcyc vs. 190 Kcyc, for initial crack length 14 mm). Numerical simulation by XFEM was verified for the BM, as shown in more details in [29], and then applied in the case of panels with stringers, including the option with welded clips. In all three cases, the BM plate, a plate with four stringers, and a plate with four stringers and additional clips with a maximum tension force of 115 kN, were applied on one end of the plate while the other was constrained (fixed), as shown in Figure 7. The stress ratio R = 0.1 used in calculations was identical to that maintained in the fatigue experiments with plates. The frequency of the applied force was 3 Hz. The XFEM model is shown in Figure 8.

**Figure 9:**
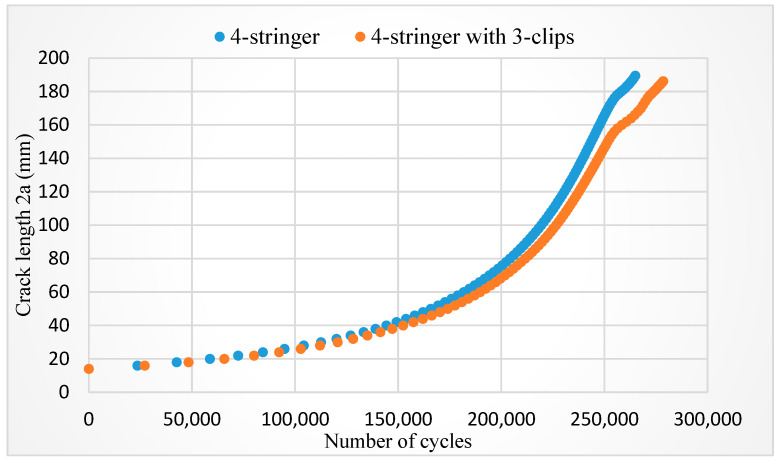
Crack length vs. number of cycles for P + 4S and P + 4S + 3C [34].

One should keep in mind the relatively large difference between experimental and numerical results, even in the case of the BM (190 Kcyc vs. 169 Kcyc), and especially in the case of the stringer panels (422 Kcyc vs. 265–290 Kcyc). As discussed in more details in [34,38,39], besides the effect of the FE mesh (290 Kcyc for element size 4 mm, 265 Kcyc for element size 1 mm), and the possible effect of coefficients C and m, the main reason for this difference could be the effect of the welded joints between the panel and stringers, which was not considered in the numerical simulation. Nevertheless, the focus of this analysis was the effect of welded clips, which was obtained by the comparison of numerical results using the same FE mesh (size 1 mm). This comparison indicated an improvement of fatigue life for only 5% of cases (278 Kcyc for panels with stringers and clips, and 265 Kcyc for panels with stringers only, Figure 9), which is certainly not worth additional welding with potential damage to the BM, as also discussed in [29,33,34]. 

## 5. Case Study 4—Riveted vs. Welded Wing Spar 

**Figure 10:**
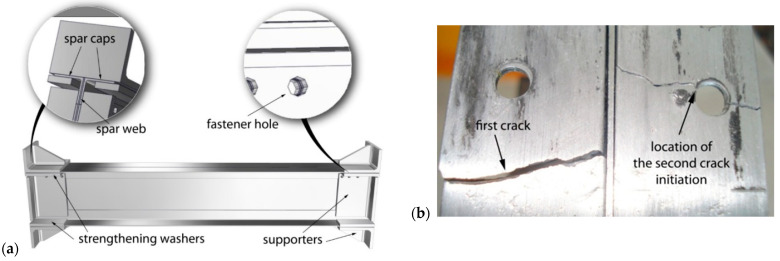
(**a**) Wing spar and supporting elements, (**b**) damaged riveted spar [35].

As the main load-carrying member of the aircraft wing, a spar is usually of an I-beam shape, made of a thin shear panel (web) and flanges (caps) at the top and bottom (Figure 10a), assembled by rivets [35,40,41,42]. A typical problem with wing spars, especially in light aeroplanes, since they have only one per wing, is fatigue crack initiation from the most severe stress concentration on the spar bottom cap, typically a fastener hole, as shown in Figure 10b. A crack can grow unnoticed, first in the spar cap, and then in the spar web, potentially leading to the failure of an aeroplane if not detected and repaired [30]. Furthermore, the design of the wing spar does not offer much of a choice in preventing this scenario. Probably the only remaining option is to use welded joints instead of riveted wing spars, to improve fatigue life in the case of cracking, as described in [35,40,41,42]. 

Experiments were performed on a riveted wing spar, made of AA2024-T3 [40], using a home-made experimental device with the minimum/maximum bending force of 391/2028 N, producing high tensile stress in caps. The first crack appeared after only 8542 cycles at the edge of the left cap, started to grow towards the spar web, and then changed its direction and continued along the cap at an angle of 90° with respect to the original direction, Figure 10a. After 39,450 cycles, the second crack began forming at the fastener hole on the right cap, as shown in Figure 10b. Cracks then continued to grow below the strengthening washers, but were not visible in up to 58,520 cycles, when the test was stopped since it looked like spar failure occurred.

**Figure 11:**
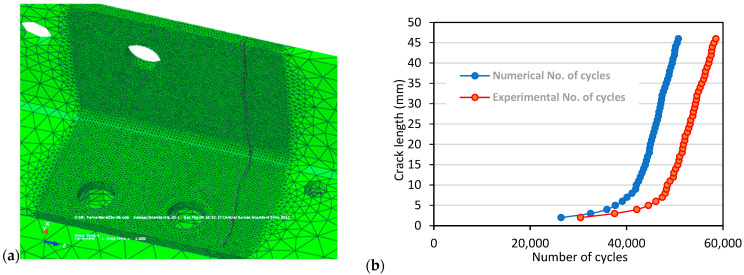
Numerical simulation of riveted spar, (**a**) crack growth, (**b**) crack length vs. number of cycles [35].

Afterwards, numerical simulation by XFEM was carried out, as described in [35,40,41,42], in two phases, first by modelling the riveted spar, and then by modelling the welded spar of the same geometry. The load used in all crack growth simulations (for both integral and differential spars) was a narrow-band random loading with a maximum displacement of 3 mm applied at one end of the spar. The other end of the spar was constrained. This displacement was identical to displacement maintained in experiments with the differential spar. The frequency of the applied displacement was 3 Hz. The initial crack was positioned at the edge of the left upper cap zone, as shown in Figure 11a, to simulate the experiment as closely as possible. A comparison of the experimental and numerical results is shown in Figure 11b, in the form of crack length vs. number of cycles, revealing excellent agreement, since the difference was very close to the number of cycles obtained by the experiment carried out to initiate the crack, which was not modelled by XFEM. However, the crack path on the vertical spar wall did not completely match the path observed in the experiment, which was attributed to local material effects being stronger than the geometry itself.

**Figure 12:**
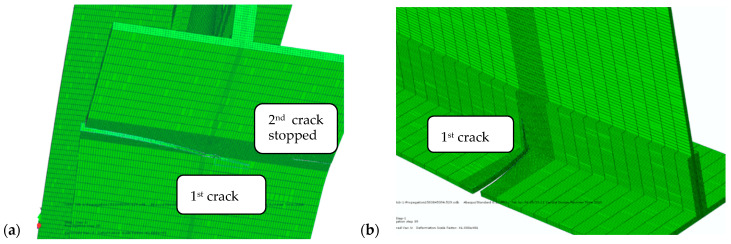
Crack tips after 35 cycles, (**a**) the cap—1st and 2nd one, (**b**) the web—1st one [35].

XFEM modelling of the welded spar, with dimensions and mass equivalent to the riveted spar, included the initial penny-shaped cracks inserted at the left and right edge of the spar cap, as described in more details in [40,41,42]. The path of the first crack on the spar was not straight, but slightly curved (Figure 12), while the second crack did not propagate after the eighth step; deformation of the spar caused by the first crack literally “closed” the second crack. The first crack then split and continued to grow simultaneously in the spar (1st front, Figure 12a) and in the web (2nd front, Figure 12b). An improvement in the number of cycles was significant, from 50 Kcyc to more than 200 Kcyc for the same geometry and mass, and up to 2 Mcyc after optimisation of the welded spar, as shown in [42].

## 6. Case Study 5—Fatigue Crack Growth Resistance of SA-387Gr. 91 Steel Welded Joints

The effects of different welded joint zones on fatigue crack growth were not considered, so far, from the point of view of different Paris law coefficients, *C* and *m*. There are two major reasons for this which are as follows: the first one, it is not a simple task to determine coefficients *C* and *m* for different welded joint zones (BM, WM, HAZ, including subzones, CG and FG); and the second one, neither ABAQUS nor ANSYS provide an option to define different values for *C* and *m* in different material zones. In this case study, experiments were performed to determine coefficients C and m for different zones of the WJ made of A387 Gr. 91 steel [36] and will be briefly described. Similar experiment were performed using Nionikral 70 steel, as shown in [43]. 

**Figure 13:**
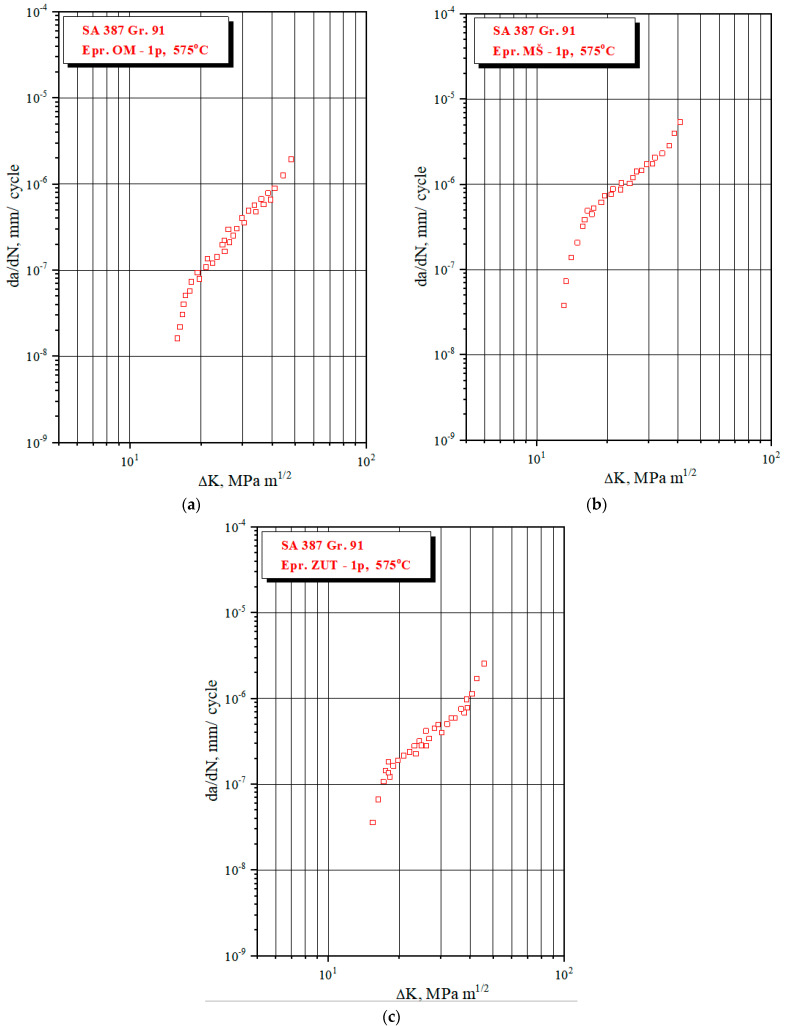
Fatigue crack growth rate vs. ΔK diagrams for 575 C, (**a**) BM, (**b**) WM, (**c**) HAZ [36].

In the case of A387 Gr. 1 steel, experiments were performed for C(T) specimens for each WJ zone, both for mechanical properties and coefficients *C* and *m* [31]. Results for the tensile properties are shown in Table 1, whereas fatigue data in the form of da/dN vs. ΔK diagrams, are shown in Figure 13. Paris coefficients *C* and *m* in are determined according to the E647 standard [29] and are also shown in Table 3.

**Figure 14:**
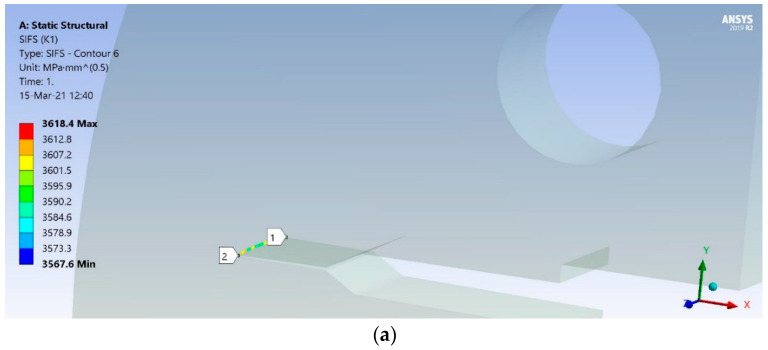

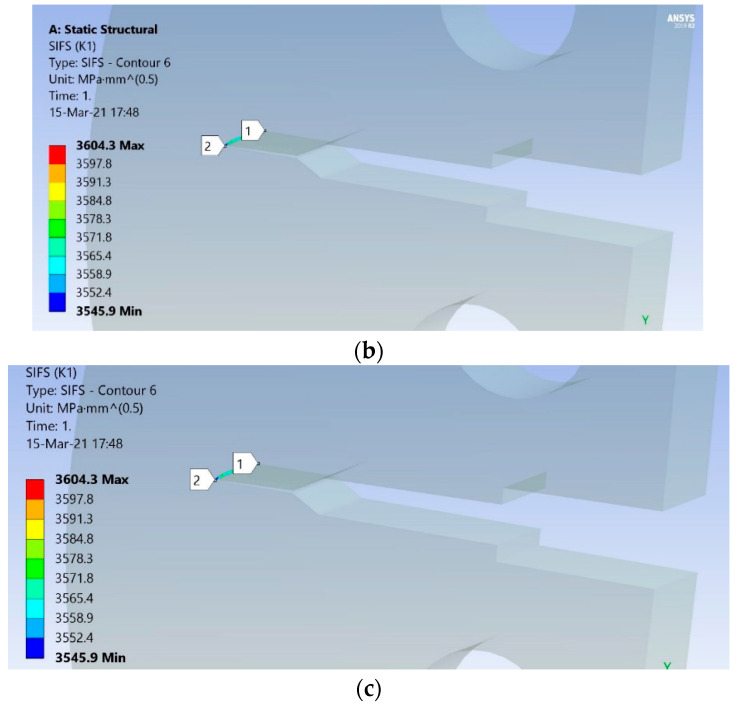
SIF distribution around the fatigue crack tip (5 mm length) for (**a**) BM, (**b**) WM, (**c**) HAZ [36].

**Figure 15:**
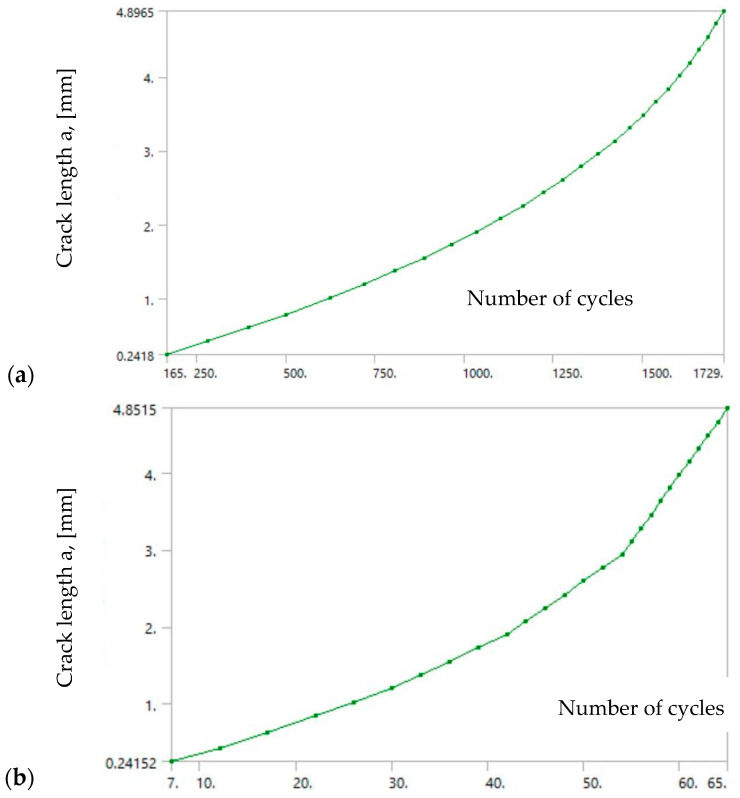

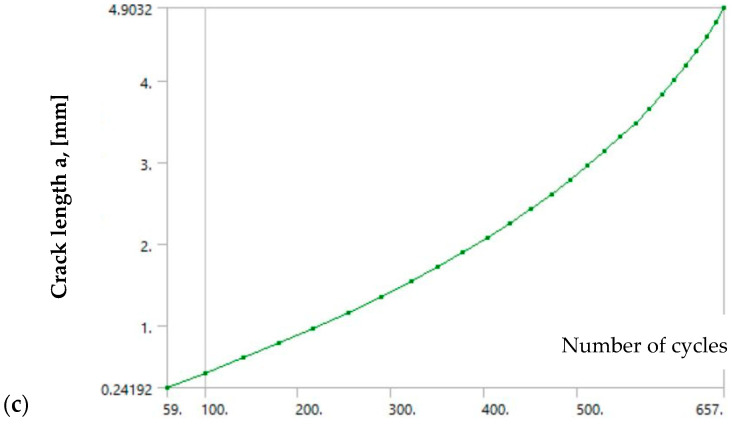
Crack length vs. number of cycles diagrams for (**a**) BM, (**b**) WM, (**c**) HAZ [36].

The numerical simulation was performed by XFEM in ANSYS with all data (FE mesh, loads, boundary conditions) defined in accordance with the experiment as is presented in [36]. Here, only the results for all three zones of the WJ are shown in Figure 14 for SIF, and in Figure 15 for the crack length vs. number of cycles.

As one can see from the a-N diagrams shown in Figure 15, the BM has the highest fatigue resistance, reaching a total of 1729 cycles, almost three times as much as the HAZ (657 cycles), while the WM had a significantly lower number of total cycles, only 65. Therefore, it is obvious that a crack initiated in the weld metal would quickly lead to failure due to fatigue. 

## 7. Case Study 6—Integrity and Life Assessment of Welded Joints Made of Micro-Alloyed High-Strength Steels Under Static and Dynamic Loading 

**Figure 16:**
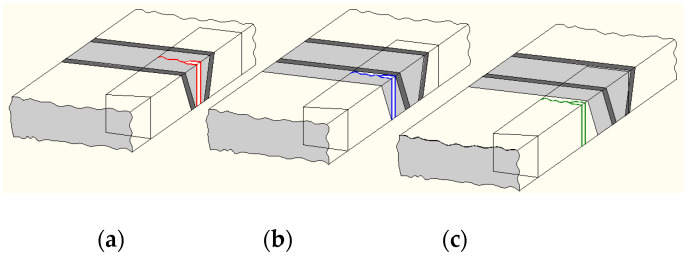
Crack tip location in Charpy specimens, (**a**) WM, (**b**) HAZ, (**c**) BM [37].

To overcome the problem of modelling WJs in ABAQUS and/or ANSYS as heterogenous materials with different values of coefficients C and m, an innovative approach has been introduced in [37] and applied in various papers published thereafter [44,45,46]. The determination of coefficients C and m was performed in accordance with previous experience [36,43], so Charpy specimens were prepared from different zones of a welded joint, as shown in Figure 16.

The welded joint used in this research was made of HSLA steel P460NL1 by SMAW, with VAC 65 used as the filler material [40]. Tensile properties of the BM, WM, and HAZ are shown in Table 4. Yield stress and tensile strength of the heat affected zones were determined based on the hardness of the regions through which the crack propagated and were around 570 MPa and 830 MPa. This was conducted in accordance with empirical formulas which are commonly used for this group of steels (HSLA).

**Figure 17:**
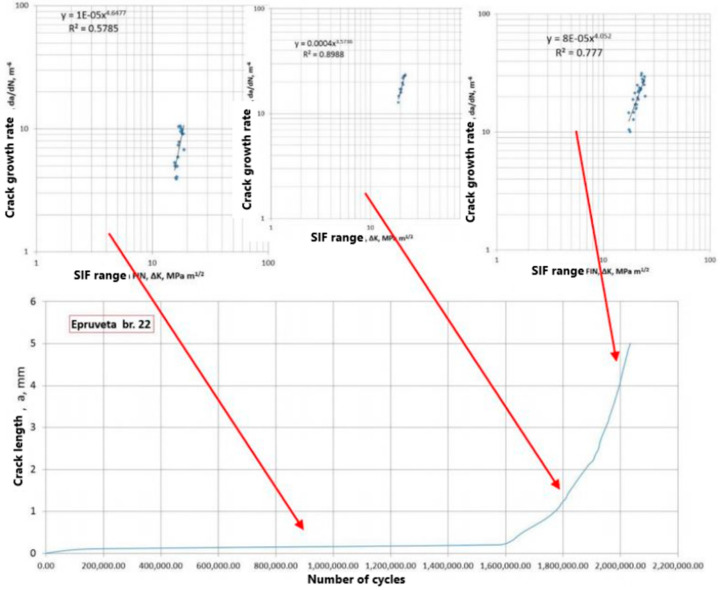
Crack length vs. number of cycles for specimen no. 22, with different slopes in different WJ zones [37].

The experiment involved fatigue testing of Charpy specimens on RUMUL Fractomat 7609/213, up to a crack length of 5 mm. Eight specimens were tested, divided into four groups, as described in [36,46]. For the analysis here, two specimens were selected, no. 5 and no. 22, both with the notch in the root side, taken from the opposite sides of the WJ. Since our focus here is the innovative procedure for numerical simulation of FCG in the WJ, we will skip other experimental details, including material specification and tensile properties, as well as the experimental determination of coefficients C and m, which are described in [37,46], except the results for specimen no. 22, which was consistent through all zones of the WJ due to FCG, as one can see in Figure 17.

**Figure 18:**
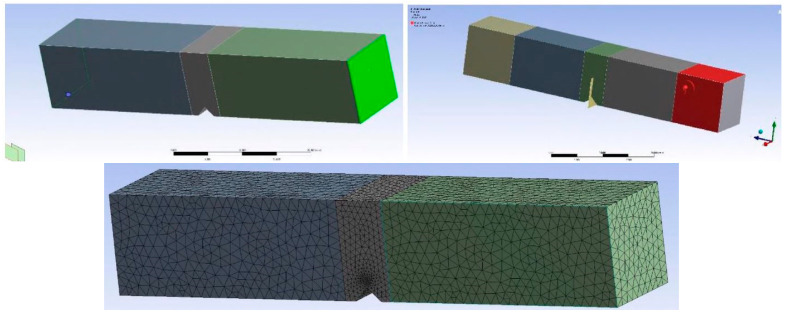
Boundary conditions and FE mesh [37].

**Figure 19:**
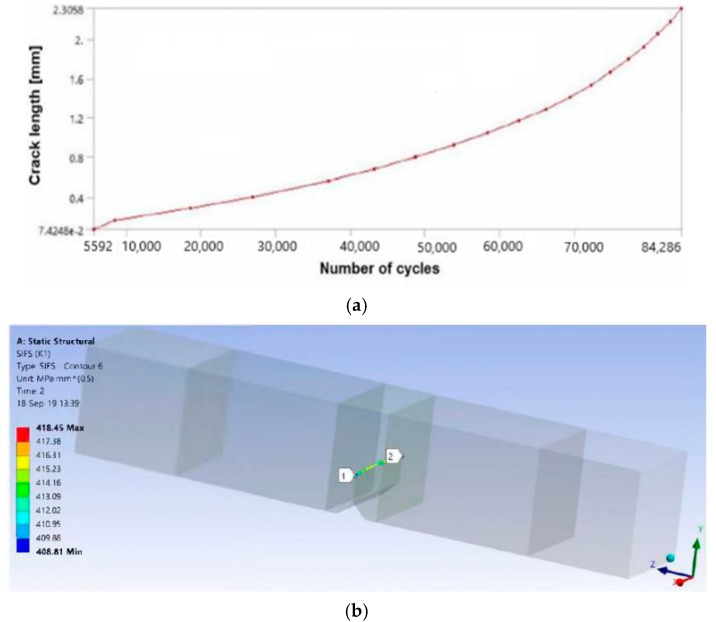
Results for (**a**) crack length vs. number of cycles, (**b**) SIF distribution [37].

Five numerical models were created in ANSYS R19.2 software to enable the calculation of the stress intensity factors and the number of cycles to used reach the critical crack length. Boundary conditions and loads were defined as was the case in real experiments—one specimen was end-fixed and the other were subjected to a bending moment (values of bending moments corresponded to the ones from the actual equipment used for fatigue simulation), as shown in Figure 18, together with the FE mesh. Three models were made for the specimen no. 22 with crack growth from the BM, and then through the HAZ and WM. Results are shown in Figure 19 and in Table 5 and Table 6.

Table 5 indicates the good comparison of the numerical and experimental prediction of the number of cycles, after certain changes were made to the Paris coefficient values, as explained in [46,47]. A similar case is with the C and m values, as shown in Table 6. In any case, numerical models presented here proved the possibility of simulating fatigue crack growth through several different welded joint regions, considering their different tensile and fatigue properties. The main cause of these errors (differences) was due to the fact that the numerical simulations had to include an initial crack, whereas in the experiment this was not the case. 

## 8. Discussion

The fracture behaviour of the mismatched welded joints turned out to be a bit surprising in the two analysed cases. Namely, as it was shown for cracks in the HAZ of the overmatched WJ and cracks in the WM of the undermatched WJ, material heterogeneity in both cases provides additional resistance to crack growth in comparison to the HAZ and the WM alone. In the case of the crack tip located at the HAZ of an overmatched WJ, the plastic strain was shifted toward a more ductile base material regardless of the location of the crack tip (FG or CG HAZ), although mechanisms are different. Also, a different way of protecting the undermatched WJ is provided by the simple redistribution of plastic strain in the case of the crack tip in WM, since it also starts developing in the BM before WM fails [42]. This mechanism is acting only if the undermatched WJ can sustain plastic straining at a sufficient level. One can speculate that this is not the case for a crack in the WM of an overmatched WJ, which is probably the most critical case, since it is not likely that such a WJ is capable of sustaining even a small amount of plastic strain, contrary to the undermatching effect. On the other hand, if the crack tip would be in the HAZ of an undermatched WJ, it is most probable that the shielding effect due to material heterogeneity would act in the same or similar way as that for an overmatched WJ.

From the point of view of the capability of FEM to simulate the fracture behaviour of the statically loaded WJ, it was proved in [17,18] for both over- and undermatched cases. In the first case [17], the agreement between numerical and experimental results was excellent. In the second case [18], the difference between experimental and numerical results for the J integral were not significant, although it showed an increasing tendency with increasing load. This was attributed to static crack growth, which was not modelled by FEM in this case.

Fatigue behaviour of WJs was analysed in four cases, two of them when material heterogeneity was not considered and two of them when it was taken into account. In the first two cases it was the geometry effect that was accounted for, as it was very beneficial for the wing spar and not beneficial enough for the stringer panels. More specifically, as it was shown in the case of the welded wing spar, the number of cycles until failure significantly increased in comparison to the riveted spar of the same geometry and mass, more so in the case of optimised geometry. Contrary to that, 5% of the increase in the number of cycles in the case of additional welded clips in a stringer panel does not justify the additional costs and risk of material damage due to welding.

The two final cases analysed in this paper have shown all the complexity of the numerical simulation of fatigue crack growth through different zones in the WJ. As it turned out, two major problems are still to be solved. One is the determination of coefficient C and m for different zones (BM, WM, HAZ), and the other is the missing option in ABAQUS and ANSYS for heterogenous properties regarding fatigue crack growth. To tackle the second problem, an innovative procedure is introduced, but the first one is still unresolved because the additional adjustments of coefficients C and m were necessary. It seems that only a kind of calibration of C and m values, using numerical simulation of an experiment with the WJ can provide an accurate prediction for this WJ, whereas individually determined coefficients C and m cannot. 

## 9. Conclusions

Based on the presented results, one can conclude the following:

- The numerical simulation of the fracture behaviour of the welded joints is well advanced and presents a versatile tool for the detailed analysis of the mismatching effect due to material heterogeneity.
- The agreement between numerical and experimental results was good in both cases regarding static fracture, with insignificant increasing differences with increasing load due to the absence of static crack growth simulation.
- The numerical simulation of fatigue crack growth in welded joints has also become a reliable tool for predicting the number of cycles to failure, but it still has to be improved in respect to the determination of coefficients C and m in different zones, so that the Paris law can be successfully applied to heterogeneous materials.
- The innovative procedure for the determination of C and m in different WJ zones was successfully applied to simulate fatigue crack growth through different zones of the WJ and evaluate fatigue life more precisely than when WJ is treated as homogeneous material.

## Figures and Tables

**Table 1:** Chemical composition of SM 80P steel and of MAW and SAW weld metals [18].

Element %wt		C	Si	Mn	P	S	Cu	Cr	Ni	Mo	V	B	Ceq
SM 80P		0.10	0.30	0.90	0.01	0.008	0.24	0.48	1.01	0.47	0.03	0.0016	0.5
Weld metal	MAW	0.06	0.53	1.48	0.011	0.005	-	0.24	1.80	0.43	-	-	-
	SAW	0.07	0.37	1.87	0.01	0.011	-	0.44	0.13	0.73	-	-	-

**Table 2:** Tensile properties of SM 80P steel and of weld metal for SMAW and SAW [18].

Material	Direction	Tensile Properties		
		Y.S. [MPa]	T.S. [MPa]	Strain [%]
SM 80P	Rolling	Min. 755	Min. 804	Min. 24
	Cross rolling	Min. 755	Min. 795	Min. 22
Weld metal	SMAW	722	810	22
	SAW	687	804	23

**Table 3:** Mechanical properties of welded joint regions.

Welded Joint Zone	Yield Stress, R_p0.2_, MPa	Tensile Strength, R_m_, MPa	Strain, A, %	Coeff. C	Coeff. m
Base material	325	495	35.0	6.00 × 10^−12^	3.22
Weld metal	495	605	21.0	2.59 × 10^−11^	3.46
Heat affected zone	495	605	21.0	2.60 × 10^−10^	2.20

**Table 4:** Mechanical properties of welded joint regions.

Welded Joint Zone	Yield Stress, R_p0.2_, MPa	Tensile Strength, Rm, MPa	Strain, A, %	Coeff. C	Coeff. m
Base material	460	607	35.0	6.00 × 10^−12^	3.22
Weld metal	460	690	21.0	2.59 × 10^−11^	3.46
Heat affected zone	570	830	21.0	2.60 × 10^−10^	2.20

**Table 5:** Comparison between numerical and experimental number of cycles for all 5 models.

Model	Number of Cycles, Numerical	Number of Cycles, Experimental	Error
PM 22	1,412,500	1,613,000	12.4%
HAZ 22	317,500	375,000	15.3%
WM 22	40,450	45,000	10.1%

**Table 6:** Comparison of new Paris coefficient values with the experimental ones.

C Experimental	C Numerical	Ratio Between C_exp_ and C_num_)	m Experimental	m Numerical	Ratio Between m_exp_ and m_num_
1 × 10^−11^	3 × 10^−13^	33.3	4.65	3.8	1.22
4 × 10^−11^	2.63 × 10^−11^	1.52	3.54	3.05	1.16
8 × 10^−11^	6.6 × 10^−12^	12.12	4.05	3.5	1.16

## Abbreviation

*a*	Crack length
*A*	Strain
BM	Base metal
*C*	Paris law coefficient
CGHAZ	Coarse-grained heat affected zone
*F*	Force
FCG	Fatigue crack growth
FEM	Finite element method
FGHAZ	Fine-grained heat affected zone
HAZ	Heat affected zone
HSLA	High-strength low-alloyed steel
*J*	J integral
Δ*K*	Stress intensity factor range
LSC	Large surface crack
*m*	Paris law exponent
*N*	Number of cycles
R_m_	Tensile strength
R_p0.2_	Conventional yield stress
SAW	Submerged arc welding
SIF	Stress intensity factor
SMAW	Shielded manual arc welding
SSC	small surface crack
WJ	Welded joint
WM	Weld metal
XFEM	Extended finite element method
